# Job satisfaction, work stress, and turnover intentions among rural health workers: a cross-sectional study in 11 western provinces of China

**DOI:** 10.1186/s12875-019-0904-0

**Published:** 2019-01-14

**Authors:** Jinlin Liu, Bin Zhu, Jingxian Wu, Ying Mao

**Affiliations:** School of Public Policy and Administration, Xi’an Jiaotong University, Xi’an, China

**Keywords:** Turnover intention, Job satisfaction, Work stress, Rural health worker, Mediating effect, Western China

## Abstract

**Background:**

Health workforce turnover remains a global concern, particularly in rural and remote areas. Western rural areas are the least developed in China, where it faces the serious challenge on the rural health worker (RHW) management. This study aimed to investigate job satisfaction, work stress, and turnover intentions of RHWs, and to explore prominent factors associated with turnover intentions of RHWs in rural western China.

**Methods:**

From June to September 2013, based on a three-stage random sampling method, a cross-sectional survey was conducted among RHWs in 11 western provinces in China. A brief, structured questionnaire filled in by RHWs was used for data collection. A total of 5046 RHWs participated in the study. The response rate was approximately 93.1%. Exploratory factor analyses, Pearson’s chi-squared tests, one-way ANOVA, binary logistic regression analyses, and mediating effect tests were performed for data analyses.

**Results:**

Approximately 29.1% of the 5046 RHWs indicated turnover intentions. Most of the RHWs disclosed low educational levels, income levels, and professional technical titles. The RHWs expressed slight job satisfaction (mean 3.20) and moderate work stress (mean 3.22). Age, income, medical institution, and job satisfaction (i.e., organizational management, reward, and occupation satisfaction) were significant predictors of the RHWs’ turnover intentions. The RHWs, who were younger (less than 41 years), receiving an income of $326.8–$490.1 per month, working in township hospitals, and having low job satisfaction, were more likely to have turnover intentions. Work stress had an indirect and positive effect on RHWs’ turnover intentions. Job satisfaction weakened the positive effect of work stress on turnover intentions of RHWs by playing a total mediating role. Reward satisfaction was the strongest mediator.

**Conclusions:**

The turnover intentions of RHWs in western China are significantly associated with job satisfaction, work stress, age, income, and medical institution. Appropriate strategies should be implemented to improve RHWs’ job satisfaction and reduce their work stress. Meanwhile, providing more attractive wages and non-monetary support, improving working conditions, etc. could be effective to reduction in RHWs’ turnover intentions.

**Electronic supplementary material:**

The online version of this article (10.1186/s12875-019-0904-0) contains supplementary material, which is available to authorized users.

## Introduction

No health without a workforce has been a universal truth [1]. However, shortage and misdistribution of qualified health workforce have become global concerns affecting nearly all counties, especially the rural and remote areas. The process of attracting additional health workforce to work in these areas has drawn considerable attention from policymakers and researchers. Besides, the retention of qualified health workers who have been working and living in these rural and remote areas has been considered another primary issue that requires attention. Various risk factors, such as low salary, lack of opportunities for career advancement and continuing educational training, and inadequate working and living conditions, etc., have been addressed [2–4]. In response to these challenges, the World Health Organization (WHO) has proposed a series of evidence-based global policy recommendations to increase access to health workers in remote and rural areas through improved retention [5]. Currently, health workforce turnover is a costly problem, which exists not only in remote rural areas but also in developed urban regions.

Turnover intention is an effective proxy predictor of the actual turnover behavior of health workers [6]. A variety of predictors influencing the turnover intentions of health workers have been identified. Job satisfaction is an important predictor [7] and it has been demonstrated a significant inverse association with turnover intentions of health workers in previous studies conducted in Ghana [8], Belgium, Germany, and the Netherlands [9], United States [10], China [11–13], Alabama [7], England [14, 15], and Scotland [16]. Work stress is another key predictor [17]. A robust positive association between work stress and turnover intention has been manifested among health workers in Alabama [7], Belgium, Germany, and Netherlands [9], China [11, 18, 19], United States [20], Jordan [21], South Korea [22], 10 European Countries [23], England [15], and Scotland [16]. Meanwhile, some sociodemographic characteristics, such as age, marital status, and education, might have affected the turnover intentions of health workers [9]. In addition, several studies have found that work stress is significantly and inversely related to job satisfaction among health workers in the United States [10, 24], Singapore [25], Belgium [26], and Greece [27]; and job satisfaction has been identified as a significant mediator in the relationship between work context factors (i.e., task content, social environment, and supervisor relationship) and turnover intentions among nurse anesthetists [28]. A study by Kuo et al. also found that job satisfaction played a significant mediation role in the relationship between work stress and turnover intention among nurses in Taiwan [29].

China is confronting these severe health workforce challenges, particularly in western rural areas, which are generally the least developed. Approximately 43.9% of the Chinese population live in rural areas [30], with 37.7% of Chinese health workers employed in rural hospitals [31]. Hospitals in China were divided into three levels, including the first-, second-, and third-level. The higher the level, the better the hospital and the better the medical resources; therefore, although work stress of health workers in high-level hospitals was higher because of more patients than those working in low-level hospitals, the working conditions were better, such as better salary and welfare treatment, more opportunities for training and learning advanced medical technology and equipment, and more career development opportunities, etc. [32, 33] Meanwhile, health workers in urban hospitals had better living conditions and social resources than those working in rural hospitals [33]. In addition, all the third-level hospitals were located in urban areas and rural areas only had the first- and second-level hospitals, so health workers working in low-level and rural hospitals had low job satisfaction and more turnover intentions [32, 33]. A prior study (Meng et al.) conducted in China reported that approximately 8% of primary health care workers left their current hospitals in 2015, among which half had moved to high-level hospitals [34]. And that was why majority of medical graduates in China competed to be employed in urban hospitals [35]. However, no study has been found to analyze turnover intentions, job satisfaction, and work stress of rural health workers (RHWs) in western China, as well as the mediating effect of job satisfaction.

Based on a cross-sectional survey with a large sample size of rural health workers in western China, this study aimed to explore: 1) RHWs’ turnover intentions; 2) job satisfaction and work stress; 3) the effects of job satisfaction and work stress on turnover intention; 4) the mediating effect of job satisfaction; and 5) other determinants.

## Methods

### Study design and participants

This cross-sectional study was a part of the collaborative research project, “*Situational Analysis and Policy Evaluation of Deployment and Retention of Human Resources for Health in Rural Western China*,” funded by the China Medical Board (CMB) and technically supported by the WHO [36].

The survey was conducted among RHWs in 11 western provinces in China (i.e., Gansu, Guangxi, Kweichow, Inner Mongolia, Ningxia, Qinghai, Shaanxi, Sichuan, Tibet, Xinjiang, and Yunnan). In this study, the RHWs were the health workers working in medical institutions located in the county. Meanwhile, the health workers are health-care providers including doctors, nurses, pharmacists, etc. Health-care managers and support workers were not involved. A total of 11 research teams from the 11 provinces participated in the survey coordinated by Xi’an Jiaotong University.

A three-stage randomized sampling method was used. The sample sizes for counties, medical institutions, and health workers were negotiated and determined by all the co-PIs of the CMB-funded project and experts from the WHO under consideration of the survey duration and budget. First, in every province, three counties were randomly selected according to the GDP per capita ranking of all counties. Second, in each county, there were four county-level medical institutions, including one County General Hospital (CGH), one Traditional Chinese Medical Hospital (TCMH), one Maternity and Child Healthcare Hospital (MCHH), and one Center for Disease Control and Prevention (CDC), and several Township Hospitals (THs). We selected three THs randomly and invited all the four county-level medical institutions to participate in the survey. Third, 50 health workers (if available) were selected randomly from each CGH, TCMH, and MCHH, and 30 health workers (if available) were selected randomly from each CDC because of fewer health workers working there than other three county-level medical institutions. Meanwhile, fewer health workers were working in THs, so we invited all of them in each TH to participate. Approximately a total of 6000 RHWs were selected; however, only the RHWs who were willing to participate in the survey answered the questionnaires. Lastly, 5584 questionnaires were collected. The response rate was approximately 93.1%. Of which, 90.4% (5046) were retained in the study after the data were incorporated and checked by Xi’an Jiaotong University. Five hundred thirty-eight questionnaires were excluded because of missing values regarding turnover intentions or other important variables.

### Data collection and variable measurement

A brief, structured questionnaire was developed for data collection. The completed questionnaire was developed initially by the research team of Xi’an Jiaotong University according to the research objectives of the CMB-funded project and referring to some useful instruments; however, we did not use them directly but made some adjustments according to the practical situation of rural medical institutions. Then, each of other ten research teams from ten western provinces in China validated the questionnaire by group discussion and expert consultation. Some research teams also made small-scale pre-surveys on RHWs to validate the logic and rationality of the questionnaire. Finally, all research teams revised and completed the questionnaire together. During this process, Dr. Fethiye Gulin Gedikg and Dr. Chunmei Wen, two experts from the WHO, provided sufficient technical support for the questionnaire design [36]. Data were collected from June to September 2013. All the questionnaires were completed by RHWs themselves. Considering the objectives of this study, we extracted only the relevant variables and data. They consisted of four sections, including general sociodemographic information, job satisfaction, work stress, and turnover intention. An additional file shows these in more detail (see Additional file 1).

Variables used to measure sociodemographic characteristics were as follows: (1) gender: female and male; (2) age was a continuous variable and was divided into three groups: < 30, 31–40, and ≥ 41 years; (3) marital status: unmarried (never unmarried) and married (ever-married); (4) education: low- (senior high school or below), medium- (secondary technical school or junior college), and high-level (bachelor or above); (5) monthly income was a continuous variable and was divided into four groups: < $163.4, $163.5–$326.7, $326.8–$490.1, ≥ $490.2; (6) technical title (The technical titles of different professions of health workers were similar and we just listed the technical titles of doctors here): medical assistant, resident, attending, associate chief, and chief physicians; and (7) medical institution: TH, CDC, MCHH, TCMH, and CGH.

Job satisfaction and work stress were measured by twenty-eight and eight items, respectively. For each item, respondents were asked as: “*please indicate how much you agree with the following statement.”* (A five-point Likert scale: strongly disagree, somewhat disagree, neutral, somewhat agree, strongly agree). Each item was scored from 1 to 5 representing *strongly disagree* to *strongly agree*. The reliability statistics results showed that Cronbach’s alphas for job satisfaction and work stress were 0.923 and 0.829, respectively, indicating acceptable scale reliability.

Exploratory factor analysis (EFA) was conducted to induce dimension reduction. The values of Kaiser–Meyer–Olkin (KMO) for job satisfaction and work stress were 0.927 and 0.823, respectively, and both *P*-values of Bartlett’s test of sphericity were less than 0.01, indicating acceptable construct validity. Based on EFAs, the job satisfaction comprised four subdomains (factors): organizational management satisfaction (OMS, it referred to satisfaction with institution’s management ability, way, and effect, interactions with managers, etc.), reward satisfaction (RS, it referred to satisfaction with the reward), occupation satisfaction (OS, it referred to satisfaction with the profession as a health worker, occupational environment, career development, etc.), and social recognition satisfaction (SRS, it referred to satisfaction with social recognition from patients, residents, etc.); and the work stress comprised two subdomains (factors): workload and negative emotion. For further calculation, the score for each factor of job satisfaction and work stress was first calculated as the mean score of related estimated items; second, the overall score of job satisfaction and work stress were calculated based on the score of estimated factors (subdomains) and their standardized values of variance contribution rates. Score of job satisfaction (total and four subdomains) and work stress (total and two subdomains) ranged from 1 to 5, indicating that a high score equated to high job satisfaction or high work stress.

Turnover intention was determined using a dichotomous question, “*Do you have the intention to quit your current job?*” answered with either yes or no.

### Statistical methods

Continuous variables (i.e., age and income) were tested for normality first by one-sample K–S tests. These variables presented an abnormal distribution and were described using the “median” and “interquartile range (IQR).” Categorical variables were displayed by “number” and “percentage.” Variables related to job satisfaction and work stress were presented by “mean” score and “standard deviation (SD).”

A Pearson’s chi-squared test was used to assess differences in proportions of sociodemographic characteristics between the RHWs with and without turnover intentions. One-way ANOVA was conducted to assess differences in the mean scores of job satisfaction and work stress between the RHWs with and without turnover intentions. *P*-values were reported.

A binary logistic regression analysis was performed to determine risk factors associated with the turnover intentions of RHWs. The independent variables selected into the binary regression models were those already statistically significant in prior univariate analyses. The results were presented as crude and adjusted odds ratio (OR) with 95% confidence interval (CI). All the significance levels were set at *P*-value < 0.05.

**Fig. 1:**
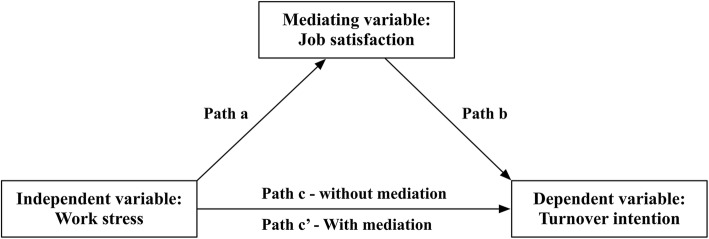
Conceptual diagram about mediating effect test

In addition, the PROCESS macro for SPSS (Model 4) was used to explore the mediating effect of job satisfaction on the relationship between work stress and turnover intentions of RHWs [37]. Bootstrapping test was used to verify the indirect effect (bootstrap samples: 5000, bootstrap CI method: bias corrected) (the conceptual diagram is illustrated in Fig. 1). This method included three steps. The first step tested the association between work stress (independent variable) and turnover intention (dependent variable) (path c). The second step tested the association between work stress (independent variable) and job satisfaction (mediator) (path a). The third step tested the model with the independent variable and mediator predicting the dependent variable, demonstrating that the mediator was associated with the dependent variable (path b) in multiple regression model. The path between work stress and turnover intention (known as path c’ in the two-predictor model) was also tested to determine whether this value was reduced to zero (total mediation) or a significant amount (partial mediation) [29, 38].

The Statistical Package for Social Science 24.0 (SPSS, IBM, Armonk, New York, USA) for MAC was used for data analysis.

### Hypotheses

The specific hypotheses were proposed in this study as follows:

- Hypothesis 1: Job satisfaction will negatively influence turnover intentions of RHWs.
- Hypothesis 2: Work stress will positively influence turnover intentions of RHWs.
- Hypothesis 3: Job satisfaction will play a mediating role in the relationship between work stress and turnover intentions of RHWs.

## Results

### Sociodemographic characteristics

A total of 5046 RHWs participated in the study. Sociodemographic characteristics are summarized in Table 1. A total of 68.0% of the respondents were female. The median age was 33 years (IQR: 27–41 years), and 39.4% were less than 30 years old. A total of 73.9% were married or ever-married. Furthermore, 71.4% attained medium-level education (i.e., secondary technical school or junior college). The median income was $359.4 per month (IQR: $294.0–$490.1), and 75.1% received an income of $163.5–$490.1 per month. Moreover, 42.3% held a technical title of resident physician. 29.9% were working in THs, and 27.5% were working in CGHs.

**Table 1:** Sociodemographic characteristics of RHWs (*N* = 5046)

Characteristics	N (%)	Turnover intention		*P*-value (χ^2^ test)
		No, *n* (%)	Yes, *n* (%)	
Gender				0.765
Female	3431 (68.0)	2428 (67.9)	1003 (68.3)	
Male	1615 (32.0)	1150 (32.1)	465 (31.7)	
Age				0.000
< 30 years	1987 (39.4)	1381 (38.6)	606 (41.3)	
31–40 years	1709 (33.9)	1175 (32.8)	534 (36.4)	
≥ 41 years	1350 (26.8)	1022 (28.6)	328 (22.3)	
Marital status				0.158
Unmarried	1316 (26.1)	913 (25.5)	403 (27.5)	
Married	3730 (73.9)	2665 (74.5)	1065 (72.5)	
Education				0.008
Low	84 (1.7)	68 (1.9)	16 (1.1)	
Medium	3603 (71.4)	2582 (72.2)	1021 (69.6)	
High	1359 (26.9)	928 (25.9)	431 (29.4)	
Income (USD, per month)				0.000
< 163.4	436 (8.6)	302 (8.4)	134 (9.1)	
163.5–326.7	1942 (38.5)	1324 (37.0)	618 (42.1)	
326.8–490.1	1849 (36.6)	1310 (36.6)	539 (36.7)	
≥ 490.2	819 (16.2)	642 (17.9)	177 (12.1)	
Technical title				0.802
Medical assistant	1704 (33.8)	1213 (33.9)	491 (33.4)	
Resident physician	2135 (42.3)	1500 (41.9)	635 (43.3)	
Attending physician	908 (18.0)	646 (18.1)	262 (17.8)	
Associate chief physician	224 (4.4)	166 (4.6)	58 (4.0)	
Chief physician	75 (1.5)	53 (1.5)	22 (1.5)	
Medical institution				0.000
TH	1507 (29.9)	1027 (28.7)	480 (32.7)	
CDC	616 (12.2)	485 (13.6)	131 (8.9)	
MCHH	794 (15.7)	584 (16.3)	210 (14.3)	
TCMH	739 (14.6)	490 (13.7)	249 (17.0)	
CGH	1390 (27.5)	992 (27.7)	398 (27.1)	

### Job satisfaction and work stress of RHWs

Table 2 displays the job satisfaction and work stress of RHWs. The average score of the overall job satisfaction of RHWs was 3.20 ± 0.55, with a degree of 64.1% (degree = real score/maximum score of the scale × 100%), indicating that the RHWs were slightly satisfied with their jobs. In the domain of job satisfaction, the RHWs reported the highest score of satisfaction for “social recognition” (3.71 ± 0.78, degree = 74.2%) and the lowest score of satisfaction in the “reward” domain (2.65 ± 0.74, degree = 53.0%). The average score of organizational management and occupation satisfaction were 3.40 ± 0.72 and 3.10 ± 0.74, respectively.

**Table 2:** Job satisfaction and work stress of RHWs (*N* = 5046)

Characteristics	Mean (SD)	Turnover intention		*P*-value
		No, Mean (SD)	Yes, Mean (SD)	
Job satisfaction	3.20 (0.55)	3.30 (0.54)	2.97 (0.51)	0.000
OMS	3.40 (0.72)	3.51 (0.70)	3.13 (0.69)	0.000
RS	2.65 (0.74)	2.76 (0.71)	2.38 (0.73)	0.000
OS	3.10 (0.74)	3.20 (0.73)	2.86 (0.71)	0.000
SRS	3.71 (0.78)	3.76 (0.78)	3.59 (0.79)	0.000
Work stress	3.22 (0.66)	3.19 (0.63)	3.30 (0.72)	0.000
Workload	3.39 (0.76)	3.37 (0.74)	3.43 (0.83)	0.020
Negative emotion	3.07 (0.77)	3.03 (0.75)	3.17 (0.82)	0.000

In terms of work stress of RHWs, the average score of the overall work stress was 3.22 ± 0.66, with a degree of 64.5%, indicating that these RHWs felt moderately stressed with work. In the domain of work stress, RHWs were stressed on workload (3.39 ± 0.76, degree = 67.8%), followed by negative emotion (3.07 ± 0.77, degree = 61.4%).

### Turnover intentions of RHWs

Twenty-nine point 1 % (1468/5046) of RHWs disclosed turnover intentions. Tables 1 and 2 show that significant differences were observed between RHWs with and without turnover intentions with respect to age, education, income, medical institution, job satisfaction, and work stress. Compared with RHWs without turnover intentions, these with turnover intentions presented a significantly higher proportion in the groups of less than 30 years, high-level education, low-level income, and working in THs. Meanwhile, they presented significantly lower job satisfaction and higher work stress than those without turnover intentions.

Table 3 presents the crude and adjusted OR with 95%CI for each variable which was significant in previous univariate analysis. After adjusting all these variables, age, income, medical institution, OMS, RS, and OS were significantly associated with RHWs’ turnover intentions. RHWs who were more likely to have turnover intentions were those who were less than 41 years, those who received a monthly income of $326.8–$490.1, and those who were working in THs. Meanwhile, the RHWs who had lower OMS, RS, and OS, were more likely to have turnover intentions, which supports Hypothesis 1. Although workload and negative emotion positively influenced turnover intentions of RHWs, the association was insignificant, which does not support Hypothesis 2.

**Table 3:** Binary logistic regression on turnover intentions of RHWs

Variable	OR _cru._ (95%CI)	*P*-value	OR _adj._ (95%CI)	*P*-value
Age				
< 30 years	1.37 (1.17, 1.60)	0.000	1.21 (1.01, 1.45)	0.037
31–40 years	1.42 (1.21, 1.66)	0.000	1.29 (1.08, 1.54)	0.005
≥ 41 years	1		1	
Education				
Low	1		1	
Medium	1.68 (0.97, 2.91)	0.064	1.46 (0.83, 2.58)	0.191
High	1.97 (1.13, 3.44)	0.017	1.72 (0.96, 3.09)	0.068
Income (USD, per month)				
< 163.4	1.61 (1.24, 2.09)	0.000	1.16 (0.87, 1.56)	0.320
163.5–326.7	1.69 (1.40, 2.05)	0.000	1.11 (0.90, 1.38)	0.327
326.8–490.1	1.49 (1.23, 1.81)	0.000	1.26 (1.02, 1.55)	0.032
≥ 490.2	1		1	
Medical institution				
TH	1.17 (0.99, 1.37)	0.060	1.38 (1.16, 1.65)	0.000
CDC	0.67 (0.54, 0.84)	0.001	0.74 (0.58, 0.94)	0.014
MCHH	0.90 (0.74, 1.09)	0.273	0.95 (0.77, 1.17)	0.633
TCMH	1.27 (1.05, 1.53)	0.016	1.15 (0.94, 1.41)	0.190
CGH	1		1	
OMS	0.46 (0.42, 0.50)	0.000	0.66 (0.58, 0.75)	0.000
RS	0.47 (0.43, 0.51)	0.000	0.58 (0.52, 0.64)	0.000
OS	0.53 (0.48, 0.58)	0.000	0.80 (0.72, 0.90)	0.000
SRS	0.76 (0.70, 0.82)	0.000	0.97 (0.89, 1.07)	0.562
Workload	1.10 (1.02, 1.19)	0.020	1.04 (0.94, 1.14)	0.472
Negative emotion	1.28 (1.18, 1.38)	0.000	1.07 (0.97, 1.17)	0.191

### Mediating effect test

**Fig. 2:**
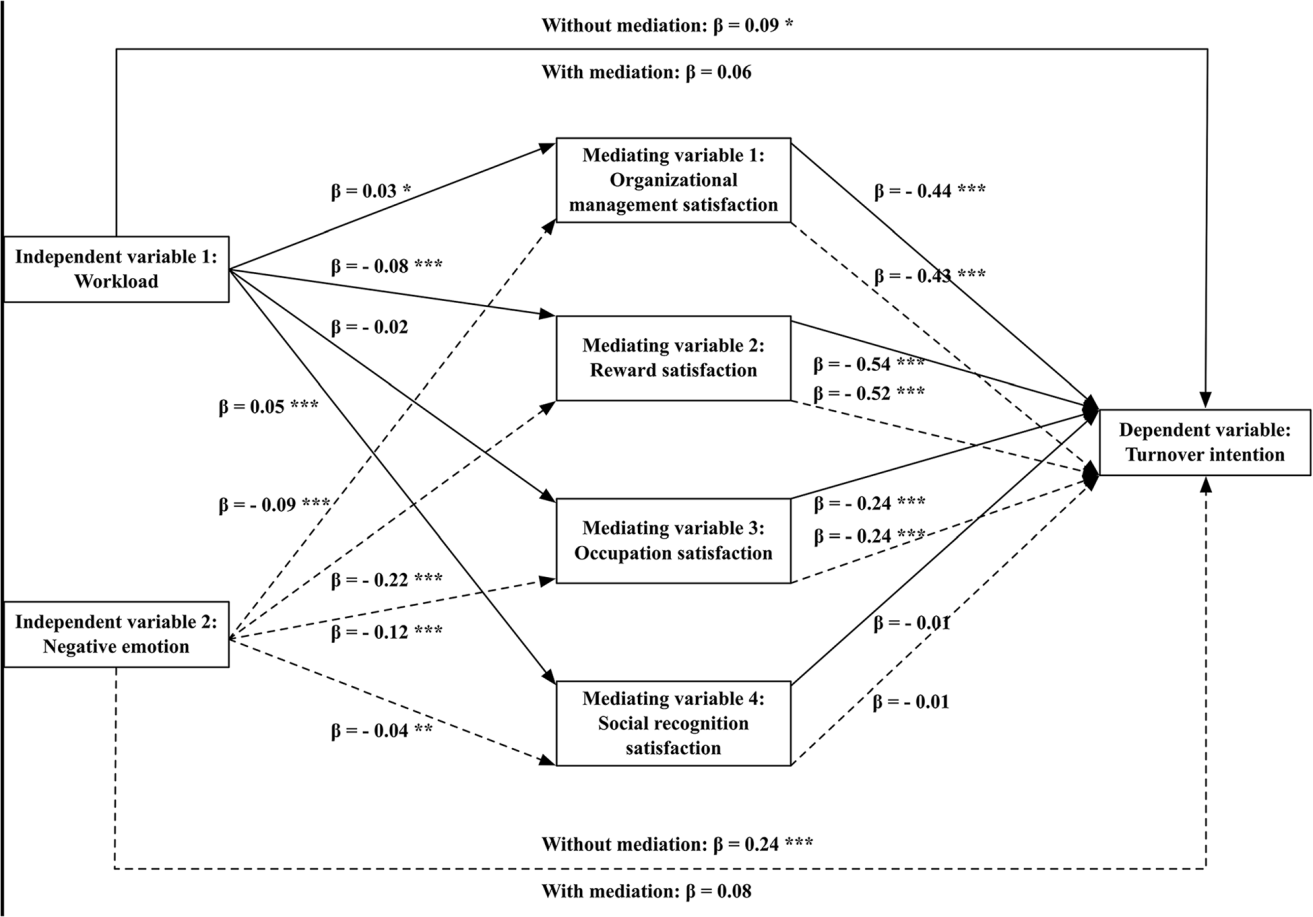
Statistical diagram about mediating effect test

Figure 2 and Table 4 show the mediating effect of job satisfaction (i.e., OMS, RS, OS, and SRS) on the relationship between work stress (i.e., workload and negative emotion) and turnover intentions of RHWs.

**Table 4:** Mediation test of job satisfaction between work stress and turnover intention

Dependent variable	Independent variable	Significance tests for effects				
			Effect	S.E.	LLCI	ULCI
Turnover intention	Workload	Total	0.0944	0.0407	0.0147	0.1742
		Direct	0.0585	0.0425	−0.0247	0.1418
		Indirect ^a^	0.0337	0.0162	0.0014	0.0654
		OMS	−0.0141	0.0069	−0.0291	−0.0022
		RS	0.0430	0.0092	0.0261	0.0621
		OS	0.0055	0.0039	−0.0009	0.0142
		SRS	− 0.0006	0.0023	− 0.0058	0.0037
Turnover intention	Negative emotion	Total	0.2436	0.0403	0.1646	0.3225
		Direct	0.0768	0.0431	−0.0077	0.1614
		Indirect ^a^	0.1858	0.0185	0.1506	0.2245
		OMS	0.0405	0.0084	0.0264	0.0602
		RS	0.1171	0.0142	0.0910	0.1473
		OS	0.0278	0.0076	0.0148	0.0446
		SRS	0.0004	0.0020	−0.0032	0.0049

^a^Significance of indirect effect was tested by Bootstrapping test presenting by Boot S.E., Boot LLCI, and Boot ULCI

The total effects of workload and negative emotion on turnover intentions of RHWs were significant and positive. After introducing the variables of OMS, RS, OS, and SRS, the direct effects of workload and negative emotion on turnover intention became insignificant and only the indirect effects were significant. Furthermore, results show that the OMS and RS played a totally mediating role in the relationship between workload and turnover intention, and they weakened the positive effect of workload on RHWs’ turnover intentions. Meanwhile, the OMS, RS, and OS played a totally medicating role in the relationship between negative emotion and turnover intention, and they reduced the positive effect of negative emotion on RHWs’ turnover intentions. All above results support the Hypothesis 3.

## Discussion

To our knowledge, this study was the first to investigate the turnover intentions of RHWs in western China and to determine the mediating effect of job satisfaction on the relationship between work stress and turnover intention. A large sample of 5046 RHWs from 11 western provinces participated in the study. It provided relevant evidence from rural western China and added to the growing body of international literature on health workers’ turnover intentions.

### Turnover intention

The results indicated that 29.1% (1468/5046) of the RHWs developed turnover intentions. China is one of the most traditional Asian countries, and the Chinese are instilled with a sense of obligation to remain in their profession [39]. RHWs who felt obliged to remain in their current profession keep themselves involved and this activity may be essential in performing their selected profession.

The percentage of RHWs with turnover intentions was lower than that in previous studies in China. Zhang et al. [40] and Fang et al. [41] reported that 52.7 and 36.8% of the rural village doctors had turnover intentions, respectively. In addition, the results varied between different countries. Dale et al. reported that 41.9% of 1192 GPs intended to leave general practice in England [15]; Bonenberger et al. found that 69% of 256 health workers in Ghana reported to have turnover intentions [8]; Ali Jadoo et al. reported that 55.2% of 576 Iraqi doctors had turnover intentions [42]; and Heinen et al. conducted a large-scale survey among 23,159 nurses from 10 European countries which showed that 9% of all these nurses intended to leave their profession and it varied from 5 to 17% between countries [23].

### Job satisfaction

Our study indicated that RHWs were slightly satisfied with their jobs. The area that they were least satisfied with was reward, especially among those who had turnover intentions. RHWs could only receive a median income of $359.4 per month, which was very low and was possibly because of the poor economic situation in western rural areas. The result was consistent with prior studies among basic-level health workers (i.e., village doctors and community health workers) from China [40, 43]. Similar results were also observed in other countries. Simoens et al. found that GPs in Scotland expressed the least satisfaction with their remuneration [16], and Bonenberger et al. reported that the score of job satisfaction with remuneration among health workers in Ghana was the lowest [8].

Meanwhile, under control of the sociodemographic variables, the organizational management satisfaction, reward satisfaction, and occupation satisfaction were significantly associated with RHWs’ turnover intentions. High job satisfaction with organizational management, reward, and occupation equated to low turnover intentions of RHWs, which was consistent with previous studies conducted in China and other countries. A study conducted by Lu et al. in China reported that job satisfaction with promotion, remuneration, current job, and superiors had a significant impact on turnover intentions of physicians [33]. Zhang et al. found that job reward and organizational management satisfaction were direct predictors of turnover intentions of physicians in China [12]. Bonenberger et al. [8], Dale et al. [15], and Ali Jadoo et al. [42] found that low job satisfaction was significantly associated with high turnover intentions of health workers in Ghana, GPs in England, and Iraqi doctors, respectively. In our study, reward satisfaction was the strongest predictor of the turnover intentions of RHWs. Steinmetz et al. reported that health care employees from Belgium, Germany, and the Netherlands with low wage satisfaction were less likely to express an intention to stay [9]. However, our study found that social recognition satisfaction was not a significant factor, which was inconsistent with previous studies. Chen et al. [11] and Yeh et al. [19] revealed that social support from patients, family/friends, and superiors was significantly associated with low turnover intentions of health workers.

### Work stress

In terms of work stress, RHWs felt moderately stressed with their work. It commonly existed among health workers, especially in China where it has the largest number of potential patients in the world [43]. In fact, patients, particularly the in-patients in China, preferred to seek health care services in high-level hospitals in cities instead of in basic-level hospitals in rural areas, causing higher workload for health workers in high-level hospitals. However, the hierarchical medical system was proposed and established with the implementation of the new health care reform in China since 2009. Patients were suggested to visit basic-level hospitals first and then proceed to high-level hospitals as referrals, if necessary. Only then could they reimburse their medical costs from the social medical insurance. Therefore, the work stress of health workers working in basic-level hospitals (including the rural hospitals) might gradually increase.

Our study indicated that there was only univariate association between work stress and turnover intentions of RHWs in this study. High work stress (workload and negative emotion) led to high turnover intentions of RHWs. This result was similar to that in previous studies. Zhang et al. found that emotional exhaustion was identified as a significant director predictor of the turnover intentions of physicians in China [12]. Another study conducted in China reported that trouble falling asleep and nerves because of work were significantly associated with physicians’ turnover intentions [33]. Workload and burnout were significantly associated with health workers’ turnover intentions in Ghana [8]. Steinmetz et al. found that working-time-related factors significantly affected health care employee’s intention to stay in Belgium, Germany, and the Netherlands [9]. Dale et al. found that volume and intensity of workload were the factors that most influenced intentions to leave general practice among GPs in England [15]. Meanwhile, Heinen et al. revealed that burnout was a very strong influencing factor associated with nurses’ intentions to leave the profession in 10 European countries [23].

In addition, when controlling other variables, the association between work stress and turnover intention in this study became insignificant. This was probably because the respondents in our study were RHWs rather than health workers working in urban medical institutions in other studies. And after testing mediating effect, the results indicated that work stress had an indirect effect on RHWs’ turnover intentions through the total mediating effect of job satisfaction including organizational management satisfaction, reward satisfaction, and occupation satisfaction. Reward satisfaction was identified as the strongest mediator. Similar results were reported by Kuo et al. that job satisfaction played the mediating role with a partial effect in the relationship between work stress and turnover intentions among long-term care nurses in Taiwan [29]. However, we didn’t found related studies conducted in other countries.

### Sociodemographic characteristics

In addition to job satisfaction and work stress, three sociodemographic characteristics, i.e., age, income, and medical institution, were significantly associated with RHWs’ turnover intentions.

First, younger RHWs were more likely to have turnover intentions than older RHWs, which was consistent with Lu et al.’s and Yeh et al.’s studies conducted in China [19, 33]. Yeh et al. found that older hospital pharmacists had lower turnover intentions and they might have experienced their career challenges and have found ways to accommodate their professional careers in their lives [19]. Similar results were found in many previous studies in other countries [9, 42, 44]. Steinmetz et al. found that young health workers were more likely to leave their jobs to seek career advancements, particularly when limited career opportunities were available within the current hospitals [9].

Second, RHWs with a lower-level income were more likely to have turnover intentions than those with a high-level income. These RHWs with a low-level income might desire to pursue a high-level income through landing a job in urban hospitals or changing professions. Income is assumed to be essential to health service delivery [9]. The results corresponded well with previous findings. Steinmetz et al. demonstrated that health care employees with a low wage were less likely to express an intention to stay in Belgium, Germany, and the Netherlands [9]. A Taiwanese study indicated that compensation (salary and bonus) was the strongest factor associated with turnovers of nurses [45]. Hinson et al. found that salary was the most important factor affecting retention of nurses in the United States [46]. Our study and Steinmetz et al.’s study [9] confirmed that both a high-level income and reward satisfaction were essential to retain health workers.

Third, the medical institution was another influencing factor. RHWs in THs were 1.38 times more likely to had turnover intentions than those working in CGHs. The working conditions of THs were worse than county- or above-level hospitals [47], thereby retaining and attracting health workers there had become slightly difficult. A similar finding from Lu et al.’s study was that physicians in low-level medical institutions (i.e., community health service center and health clinics) were more likely to have turnover intentions than those working in high-level hospitals where there were better welfare treatment, work environment, etc. [33]. However, working in CDCs, which were one of the county-level hospitals, reduced the likelihood for RHWs to have turnover intentions compared with those working in CGHs. This result might be due to a significantly higher job satisfaction and lower work stress of RHWs employed in CDCs (3.23 ± 0.52, 3.11 ± 0.58) than those working in CGHs (3.19 ± 0.57, 3.35 ± 0.66) in our study.

In addition, contrary to prior studies [9, 13, 23], our study found that gender, marital status, and education were insignificantly associated with RHWs’ turnover intentions.

### Implications

The study has several implications. As a large percentage in total health workers in China, RHW’s turnover intentions are major problems facing the rural health care system, especially in western remote region, which should attract more attention from related health service managers and policy makers. Our study highlighted the important effects of job satisfaction and work stress on turnover intentions of RHWs, especially the job satisfaction which had direct effects and played a mediating role. Thus, we recommend providing effective regulations to protect RHWs from turnover actions. It should place more emphasis on improving job satisfaction of RHWs through improving managers’ professional hospital administration and providing more opportunities for continual training and career development etc. Meanwhile, it’s essential to offer more attractive and competitive wages and benefit packages and introduce more effective payment mechanisms for RHWs and to improve their reward satisfaction. In addition, measures to reduce work stress also should be implemented under gradual implementation of new health care reform in China. Furthermore, the high-turnover-prone group of RHWs who were younger and working in THs should be paid more attention by recognizing and rewarding health workers who stay longer, improving motivation and commitment, providing non-monetary support, and improving working conditions of THs, etc.

### Limitations

Certain limitations of the study must be mentioned. First, RHWs in this study included different types of professions, such as doctors and nurses. However, it didn’t analyze the possible influence of the types of professions on RHWs’ turnover intentions. Second, the survey was conducted among RHWs in 11 provinces and the multilevel analysis should be used as a way of controlling for effects of provinces. Third, in-house instruments developed according to the practical situation of rural medical institutions in China were used to measure the job satisfaction and work stress of RHWs in this study. However, several very good instruments for these variables had been developed and validated. The measurement results on job satisfaction and work stress in our study might not be as effective as results measured by these instruments. Fourth, no comparison tests were performed for respondents and non-respondents because we did not collect the non-respondents’ information. Fifth, since the sense of obligation was important for Chinese, it should be introduced in the questionnaire and controlled for RHWs’ turnover intentions. Sixth, it did not provide definitive conclusions about the causal relationship between job satisfaction, work stress, and turnover intentions of RHWs because of the cross-sectional design. It could only show a static picture and could not circumvent the changes between the actual behavior and the stated turnover intentions of the RHWs; therefore, it could not further capture the factors affecting the actual turnover behaviors of RHWs.

## Conclusions

29.1% of RHWs in western China had turnover intentions. RHWs were slightly satisfied (3.20 ± 0.55) and moderately stressed (3.22 ± 0.66) with their work. It is concluded that RHWs’ turnover intentions were significantly associated with job satisfaction (i.e., OMS, RS, and OS), work stress (indirect effect, i.e., workload and negative emotion), and sociodemographic factors (i.e., age, income, and medical institution). Job satisfaction has a total mediating effect and it weakens the positive relationship between work stress and RHWs’ turnover intentions. The results may be useful for policy makers and health administrators wishing to retain the existing RHWs in western China. To achieve this, the appropriate policies should be developed focusing on job satisfaction and work stress, especially job satisfaction. Improving job satisfaction, especially the reward satisfaction, and reducing work stress are very important actions. A particular attention should be given to the RHWs who were younger and working in THs. This can be achieved by providing intrinsic rewards and non-monetary support, and improving working conditions of THs, etc.

## Additional file

Additional file 1: Questionnaire for rural health workers in western China. (DOCX 20 kb)

## Abbreviations

ANOVA: Analysis of variance
CDC: Center for Disease Control and Prevention
CGH: County general hospital
CI: Confidence interval
CMB: China Medical Board
EFA: Exploratory factor analysis
IQR: Interquartile range
KMO: Kaiser-Meyer-Olkin
MCHH: Maternity and child health hospital
OMS: Organizational management satisfaction
OR: Odds ratio
OS: Occupation satisfaction
RHW: Rural health worker
RS: Reward satisfaction
SD: Standard deviation
SRS: Social recognition satisfaction
TCMH: Traditional Chinese medical hospital
TH: Township hospital
WHO: World Health Organization
