# Supplementary material for: Job satisfaction, work stress, and turnover intentions among rural health workers: a cross-sectional study in 11 western provinces of China
Source: BMC Fam Pract. 2019 Jan 14;20:9. doi: 10.1186/s12875-019-0904-0 (PMC6330754; doi:10.1186/s12875-019-0904-0)
Supplement: Supplementary file 1 — Questionnaire for rural health workers in western China. (DOCX 20 kb) [file 12875_2019_904_MOESM1_ESM.docx]

**Questionnaire for rural health workers in western China**

| **1 Sociodemographic information** (*Please choose one selection only*)  1.1 Gender_____ (1) Female; (2) Male  1.2 Age_______(years)  1.3 Marital status_____ (1) Unmarried (never married); (2) Married (ever-married)  1.4 Education_____ (1) Senior high school or below; (2) Secondary technical school; (3) Junior college; (4) Bachelor or above (university)  1.5 Monthly income_____(RMB, yuan)  1.6 Technical title_____ (1) Medical assistant; (2) Resident physician; (3) Attending physician; (4) Associate chief physician; (5) Chief physician  1.7 Medical institution_____ (1) Township hospital; (2) Center for disease control and prevention; (3) Maternal and child healthcare hospital; (4) Traditional Chinese medical hospital; (5) County general hospital  **2 Job satisfaction** (*Please choose one selection only*; *1=strongly dissatisfy, 2=dissatisfy, 3=neither dissatisfy nor satisfy, 4=satisfy, 5=strongly satisfy*)   \| **NO.** \| **Items** \| **Job satisfaction** \| \| \| \| \| \| --- \| --- \| --- \| --- \| --- \| --- \| --- \| \| **1** \| **2** \| **3** \| **4** \| **5** \| \| 1 \| My income compared with colleagues in my hospital \|  \|  \|  \|  \|  \| \| 2 \| My income compared with health workers in other hospital \|  \|  \|  \|  \|  \| \| 3 \| Salary system in my hospital \|  \|  \|  \|  \|  \| \| 4 \| Income equity in my hospital \|  \|  \|  \|  \|  \| \| 5 \| Balance between my effort and salary \|  \|  \|  \|  \|  \| \| 6 \| Performance evaluation mechanism in my hospital \|  \|  \|  \|  \|  \| \| 7 \| Rationality of incentive mechanism in my hospital \|  \|  \|  \|  \|  \| \| 8 \| Respect from patients \|  \|  \|  \|  \|  \| \| 9 \| Respect from local residents \|  \|  \|  \|  \|  \| \| 10 \| Patients’ satisfaction with my services \|  \|  \|  \|  \|  \| \| 11 \| Patients’ trust in my services \|  \|  \|  \|  \|  \| \| 12 \| Level of my job’s enrichment \|  \|  \|  \|  \|  \| \| 13 \| Appreciate by leaders in my hospital \|  \|  \|  \|  \|  \| \| 14 \| The way leaders treat health workers \|  \|  \|  \|  \|  \| \| 15 \| Managerial decision-making ability \|  \|  \|  \|  \|  \| \| 16 \| Stability of my job \|  \|  \|  \|  \|  \| \| 17 \| The opportunity to play my abilities at work \|  \|  \|  \|  \|  \| \| 18 \| Mode of policy implementation in my hospital \|  \|  \|  \|  \|  \| \| 19 \| Job promotion opportunities \|  \|  \|  \|  \|  \| \| 20 \| Autonomy of my job in my hospital \|  \|  \|  \|  \|  \| \| 21 \| Professional environment \|  \|  \|  \|  \|  \| \| 22 \| A sense of achievement of my job \|  \|  \|  \|  \|  \| \| 23 \| Relationships with my colleagues \|  \|  \|  \|  \|  \|   **3 Work stress** (*Please choose one selection only*; *1=strongly disagree, 2=disagree, 3=neither disagree nor agree, 4=agree, 5=strongly agree*)   \| **NO.** \| **Items** \| **Work stress** \| \| \| \| \| \| --- \| --- \| --- \| --- \| --- \| --- \| --- \| \| **1** \| **2** \| **3** \| **4** \| **5** \| \| 1 \| There is a lot of work to do in my hospital. \|  \|  \|  \|  \|  \| \| 2 \| I’m very busy with my work every day. \|  \|  \|  \|  \|  \| \| 3 \| I often fail to finish my work on time. \|  \|  \|  \|  \|  \| \| 4 \| My work requires a lot of time and effort. \|  \|  \|  \|  \|  \| \| 5 \| I’m always nervous because of work. \|  \|  \|  \|  \|  \| \| 6 \| I am difficult to fall asleep because of work. \|  \|  \|  \|  \|  \| \| 7 \| I feel a high-level tension at work. \|  \|  \|  \|  \|  \| \| 8 \| I feel a lot of pressure at work. \|  \|  \|  \|  \|  \|   **4 Turnover intention** (*Please choose one selection only*)  4.1 Do you have the intention to quit your current job?____ (1) Yes; (2) No |
| --- | --- | --- | --- | --- | --- | --- | --- | --- | --- | --- | --- | --- | --- | --- | --- | --- | --- | --- | --- | --- | --- | --- | --- | --- | --- | --- | --- | --- | --- | --- | --- | --- | --- | --- | --- | --- | --- | --- | --- | --- | --- | --- | --- | --- | --- | --- | --- | --- | --- | --- | --- | --- | --- | --- | --- | --- | --- | --- | --- | --- | --- | --- | --- | --- | --- | --- | --- | --- | --- | --- | --- | --- | --- | --- | --- | --- | --- | --- | --- | --- | --- | --- | --- | --- | --- | --- | --- | --- | --- | --- | --- | --- | --- | --- | --- | --- | --- | --- | --- | --- | --- | --- | --- | --- | --- | --- | --- | --- | --- | --- | --- | --- | --- | --- | --- | --- | --- | --- | --- | --- | --- | --- | --- | --- | --- | --- | --- | --- | --- | --- | --- | --- | --- | --- | --- | --- | --- | --- | --- | --- | --- | --- | --- | --- | --- | --- | --- | --- | --- | --- | --- | --- | --- | --- | --- | --- | --- | --- | --- | --- | --- | --- | --- | --- | --- | --- | --- | --- | --- | --- | --- | --- | --- | --- | --- | --- | --- | --- | --- | --- | --- | --- | --- | --- | --- | --- | --- | --- | --- | --- | --- | --- | --- | --- | --- | --- | --- | --- | --- | --- | --- | --- | --- | --- | --- | --- | --- | --- | --- | --- | --- | --- | --- | --- | --- | --- | --- | --- | --- | --- | --- | --- | --- | --- | --- | --- | --- | --- | --- | --- | --- | --- | --- | --- | --- | --- | --- | --- | --- | --- | --- |
